# Influence of Different Nanomaterials on Growth and Mycotoxin Production of *Penicillium verrucosum*

**DOI:** 10.1371/journal.pone.0150855

**Published:** 2016-03-14

**Authors:** Kathrin Kotzybik, Volker Gräf, Lena Kugler, Dominic A. Stoll, Ralf Greiner, Rolf Geisen, Markus Schmidt-Heydt

**Affiliations:** 1 Department of Safety and Quality of Fruits and Vegetables, Federal Research Institute of Nutrition and Food, Max Rubner-Institut, Karlsruhe, Germany; 2 Department of Food Technology and Bioprocess Engineering, Federal Research Institute of Nutrition and Food, Max Rubner-Institut, Karlsruhe, Germany; VIT University, INDIA

## Abstract

Nanoparticles are ubiquitous in the environment. They originate from anthropogenic or natural sources or they are intentionally produced for different purposes. There exist manifold applications of nanoparticles in modern life leading unavoidably to a confrontation and interaction between nanomaterial and living organisms. Based on their wide distribution tending to increase steadily, the influence of particles based on silica and silver, exhibiting nominal sizes between 0.65 nm and 200 nm, on the physiology of the mycotoxigenic filamentous fungus *Penicillium verrucosum* was analyzed. The applied concentration and time-point, the size and the chemical composition of the particles was shown to have a strong influence on growth and mycotoxin biosynthesis. On microscopic scale it could be shown that silver nanoparticles attach to the mycelial surface. Moreover, silver nanoparticles with 0.65 nm and 5 nm in size were shown to internalize within the cell, form agglomerates in the cytoplasm and associate to cell organelles.

## Introduction

Worldwide, a quarter of the cereal based crop is contaminated by filamentous fungi and their mycotoxins and has to be rejected at the expense of the food supply of a steadily rising world population [1]. The climate change contributes to a further deterioration of the situation [2–3]. Of utmost importance are fungi which produce mycotoxins. Among the most important mycotoxins are aflatoxins (*Aspergillus*), trichothecenes and fumonisins (*Fusarium*), alternariol and tenuazonic acid (*Alternaria*) and in case of *Penicillium* ochratoxins, patulin and citrinin [4]. *Penicillium verrucosum*, which has been used throughout this study, represents a worldwide distributed fungal species. Furthermore *P*. *verrucosum* is known as wheat contaminant producing the mycotoxins ochratoxin A and citrinin.

Mycotoxins are toxic secondary metabolites of filamentous fungi whose production is induced under defined growth conditions and physiological stages of the fungal cell. There exist a large number of publications that describe the influence of various parameters and substances on growth and mycotoxin production of miscellaneous fungal species [5–7]. Nevertheless, general solutions to prevent fungal infestations are not in sight. A promising new opportunity could be the application of nano-scaled supplements e.g. as spray-impregnant for foods which are normally washed, shelled or peeled such as melons, apples or citrus fruits. Another possibility could be the use as therapeutic agent against dermatophytes. Alternatively the use as dehumidifier throughout storage may be an advantage to prolong the shelf-life of certain food commodities. For some nano-sized particles bactericidal, antifungal and antiviral effects are described [8–12].

The International Organization for Standardization (ISO) defines the term ‘nanomaterial’ as a ‘material with any external dimension in the nanoscale or having internal structure or surface structure in the nanoscale’. The term ‘nanoscale’ is defined as size range from approximately 1 nm to 100 nm according to ISO/TS 80004–1 [13]. Nanoparticles occur naturally as pigments, emulsions, volcano ashes or dusts, are produced anthropogenic like in the case of exhaust fumes, or are manufactured intentionally for the usage as additives in cosmetics, pharmaceuticals and food.

Examples for (nano)-particles used in products of daily-use and in food context are titanium-dioxide (TiO_2_) as UV-blocker in sun screens, as food-additive (E171), in toothpaste, in chewing gum, or SiO_2_ as anti-caking agent in spices and powdered sugar. (Nano)-silver is used in medical plasters, wound creams, as freezer-coating or in textiles and packaging material of certain foods, taking advantage of the bactericidal properties of the heavy metal silver. Furthermore, in the EU some metals like silver (E174) and gold (E175) and some oxides like e.g. TiO_2_ (E171) and SiO_2_ (E551) are approved as food supplements for specific applications. It has to be kept in mind that engineered nanomaterials which are used in food products underlie a statutory “duty of declaration” within the European Community. Besides, whether the respective particles are present as single nanoparticles or as agglomerates and aggregates consisting of nano-scaled primary particles remain elusive. Currently, there are discussions about the particle size of these food additives. The European Food Safety Authority (EFSA) has carried out a re-evaluation for approved food additives that were already permitted before 1/2009 [14]. Nethertheless there exist manifold applications of nanoparticles in modern life. Due to an exceptional wide variety of potential applications in technique, medicine and pharmacology the world-wide distribution of nanoparticles of anthropogenic origin shows an increasing tendency. Hence, research for nanoparticles is in principle of interest.

The aim of this study was to investigate if nanoparticles have an influence on growth and mycotoxin biosynthesis of fungi. Furthermore the study should answer the question if there exists a relationship between the measure of influence on the physiology of the fungus and the chemical composition, the amount and the size of the applied (nano)-particles.

## Material and Methods

### Fungal strain and growth conditions

*Penicillium verrucosum* (BFE575) from the culture collection of the Max Rubner-Institut was used throughout the experiments. *P*. *verrucosum* was routinely grown in triplicates on malt extract agar (MEA; 17 g/l malt extract, 5 g/l glucose, 15 g/l agar) and incubated at 25°C for 7 days in the dark. A suspension of spores has been prepared with the aid of an inoculation loop (Copan Diagnostics Inc.; Murietta, California, USA) and subsequent suspension in Tween solution (0.9 g/l NaCl, 5 g/l Tween and 5 g/l agar). The amount of spores per ml was determined by a Thoma chamber (Paul Marienfeld GmbH & Co. KG; Lauda Königshofen, Germany) using 400x magnification on a Laborlux-S optical microscope (Leica Microsystems GmbH; Wetzlar, Germany). The spore suspension was adjusted to 10^6^ spores per ml. 50 μl of the spore suspension were added to each cavity of the tissue culture plates (VWR International GmbH; Darmstadt, Germany). These plates have been prepared by first introducing 650 μl MEA-liquid-medium (17 g/l malt extract, 5 g/l glucose) in each cavity and subsequent application of the respective nanoparticles (the latter in increasing concentrations as indicated). Between the cavities of the culture plates 600 μl of sterile water was added to ensure a constant humidity and to reduce the “edge”-effect. All experiments have been performed twice in 6-fold replications for all concentrations (concentrations of applied nanoparticles see Table 1) using 48-cavity tissue culture plates. The conditions for growth were: incubation in a climatic chamber (Johnson Controls, Inc.; Norman, Oklahoma, USA) at 25°C for 7 days on an incubating shaker (model: VWR® incubating mini shaker, open hood, 230 rpm, respectively SM-30 Control rotary shaker (Edmund Bühler GmbH; Hechingen, Germany).

**Table 1:** Specifications of the applied nanomaterials (Data in S5 data set).

manufacturer and product name	used concentrations / ppm	manufacturer information				experiment associated measurements	
		"nominal size" / nm	Lot Number	Diameter TEM / nm	Z-Average / nm	Z-Average / nm	PDI
nanoComposix Non-functionalized NanoXact™ Silica (10,000 ppm)	10; 25; 100; 250; 500; 1,000; 2,500	20	DAC1478	23.2 ± 2.4	24.9	26.9 ± 0.4	0.113 ± 0.024
nanoComposix Non-functionalized NanoXact™ Silica (10,000 ppm)	10; 25; 100; 250; 500; 1,000; 2,500	50	DAC1558	50.0 ± 5.3	62.0	61.0 ± 0.5	0.062 ± 0.014
nanoComposix Non-functionalized NanoXact™ Silica (10,000 ppm)	10; 25; 100; 250; 500; 1,000; 2,500	100	DAC1510	101.7 ± 9.0	116.6	114.2 ± 0.9	0.031 ± 0.013
nanoComposix Non-functionalized NanoXact™ Silica (10,000 ppm)	10; 25; 100; 250; 500; 1,000; 2,500	200	JMW1149	202.5 ± 10.2	221.0	216.4 ± 1.2	0.015 ± 0.011
nanoComposix Citrate BioPure™ Silver (1,000 ppm)	1; 5; 25; 50; 100	5	MGM2206	4.6 ± 0.8	not reported	27.0 ± 1.3	0.589 ± 0.109
nanoComposix Citrate BioPure™ Silver (1,000 ppm)	1; 5; 25; 50; 100	50	MRL1090	51.6 ± 5.4	56.8	53.8 ± 0.4	0.155 ± 0.008
nanoComposix Citrate BioPure™ Silver (1,000 ppm)	1; 5; 25; 50; 100	200	CLF0225A	193.7 ± 21.2	193.8	187.4 ± 2.8	0.079 ± 0.014
Purest Colloids MesoSilver^®^ (20 ppm (minimum) total silver)	1; 2; 4; 6; 8	≤ 0.65	13325L131017	not reported	Size Distribution by Volume (not explicit for this LOT); peak 1: 0.62 nm (99.8%); peak 2: 1.8 nm (0.1%); peak 3: 4.72 nm (0.1%); PDI = 0.827	n.a.	n.a.

### Growth Assessment

After incubation the growth of the fungus in the respective culture medium was determined and analyzed. For documentation, pictures of the plates were taken from above and from the bottom with a Coolpix P520 camera (Nikon; Tokio, Japan). For standardized illumination a luminous plate Rex-Leuchtplatte FL-3 (Rex Leuchtplatten; Blaustein, Germany) was used. Before taking the pictures, a white balance setting was performed and focusing was set to 3.2 to obtain reproducible conditions over the whole experiments. To determine quantitative values for the growth rate, we chose the GNU Image Analysis Program GIMP 2.8.10 [15]. The principle behind this procedure has been described earlier in a similar manner [16; 17] and was chosen for two reasons. The first reason is that the fungal mycelium grows not in a radial manner in submerged culture under shaking conditions, it grows asymmetrically. Therefore, measuring the growth rate by taking two diametric measurements at right angles to each other does not appropriately reflect the growth rate as it has been discussed in Gibbs et al. [18]. The second reason is that measuring solely the diameter of the colony does not reflect increases in 3^rd^ dimension, respectively the thickness or density of the mycelium. Consequently, we select the median of the light distribution of the mycelial mat. This procedure implements the extent of the mycelial area as well as the thickness of the mycelia. The procedure could be described as followed: First, the saturation of the pictures was equalized for all pictures to reduce deviations in color. Second, single cavities were selected and the mean value, standard deviation, median and pixel-count of the brightness control were ascertained using the respective histograms. This data as reciprocal values were used as the relative growth rate.

### Extraction of Ochratoxin A and Citrinin

For determination of ochratoxin A and citrinin, a liquid/liquid-extraction using H_2_O:chloroform (50:50 [v:v]) has been carried out. The procedure could be described as follows: The entire content of each cavity was transferred to 2 ml micro-tubes with screw caps (VWR International GmbH; Darmstadt, Germany) and a stainless steel bead (6 mm; QIAGEN GmbH; Hilden, Germany) each as well. Each sample was grounded on a FastPrep-24 high-speed benchtop homogenizer (MP Biomedicals, LLC; Santa Ana, CA, USA) three times for 20 seconds at 4.5 m/s. Afterwards, the stainless steel beads were discarded, 650 μl chloroform (Carl Roth GmbH + Co. KG; Karlsruhe, Germany) were added to each extraction which were then incubated 30 min at 22 +/- 2°C on a vortex mixer at 2,000 min^-1^ (VWR International GmbH; Darmstadt, Germany). To separate the mycelial debris from the toxin extract, the tubes were centrifuged for one minute at 13,000 rpm (= 15,890 g) in a Perfect Spin 24R refrigerated micro-centrifuge (VWR International GmbH; Darmstadt, Germany) at ambient temperature. The mycelia were discarded and the chloroform extract was evaporated to dryness in a vacuum concentrator (Speed Vac, Savant Instruments; Farmingdale, NY, USA). In preparation for the HPLC the samples were reeluted in 100 μl methanol (VWR International GmbH; Darmstadt, Germany) and transferred by means of syringe filters (ø 5 mm) into HPLC vails. The extracts were stored at -20°C until further analysis.

### Detection of ochratoxin A and citrinin by HPTLC

The TLC-technique is sometimes a more eligible way to visualize the biosynthesis of a fluorescence-active compound in a sample, than absolute quantification by e.g. HPLC. The residual extracts were dissolved in 50 μl methanol and 15 μl were spotted onto pre-coated TLC-sheets (Macherey-Nagel GmbH & Co. KG; Düren, Germany). As mobile phase toluol:ethyl acetate:formic acid (60:30:10 [v:v:v]) was used. The spots were visualized under UV light at 254 nm and 366 nm on a TLC Scanner and Visualizer (CAMAG; Muttenz, Switzerland). All solvents used in the HPTLC as mobile phase were purchased from Carl Roth GmbH + Co. KG (Karlsruhe, Germany). All standards used in HPTLC and HPLC analyses were obtained from Sigma-Aldrich Co. LLC. (Taufkirchen, Germany).

### Quantification of ochratoxin A and citrinin by HPLC

Quantitative determination of ochratoxin A and citrinin has been performed by HPLC as already described in [19]. In case of silver nanoparticles the TLC technique has been favored for two reasons. First, the silver particles which remain in the mycelial extracts disturb an exact and reproducible detection by the fluorescence detector of the HPLC system. Second, the trend of the toxin biosynthesis in relation to the concentration of silver nanoparticles could be well visualized by using TLC plates. Respectively, extracts of 6 biological replicates were pooled to minimize organismal variation.

### Microscopy of mycelia containing nanoparticles

Sample preparation for microscopically examination of fungal filaments has been carried out using cavity-slides of 15 mm diameter (Carl Roth GmbH + Co. KG; Karlsruhe, Germany). The cavities were filled with sterile H_2_O and a defined piece of mycelium from the experiments was added under the aid of a pair of tweezers. The cavities were covered with cover slips (Carl Roth GmbH + Co. KG; Karlsruhe, Germany) and the samples were sealed with crystal-clear nail polish. Microscopy was carried out on an Axio-Imager microscope platform (Carl Zeiss AG; Oberkochen, Germany), enabling the photographical analysis of the preparations, which were examined at 400x-1000x magnifications, the latter with oil-immersion.

### Determination of reactive oxygen species (ROS)

For measuring the degree of total free radical oxygen species in the incubated samples the OxiSelectTM InVitro ROS/RNS Assay Kit (Green Fluorescence, (Cell Biolabs, Inc., San Diego, USA) was used according to the manufacturer's recommendations.

### Statistical analyses

Determination of statistical significance (p-value) has been done using SigmaPlot 12.3: OneWay ANOVA.

### Applied Nanomaterial

Nanoparticles were purchased from the following suppliers: MesoSilver^®^ (Purest Colloids, Inc.; Westhampton, NJ, USA); Citrate BioPure™ silver spheres and non-functionalized NanoXact™ solid silica spheres exhibiting different sizes (NanoComposix; Prague, Czech Republic). For further information regarding e.g. sizes and concentrations of the particles see Table 1. NanoComposix specifies the particle sizes on the datasheets as determined by transmission electron microscopy (diameter TEM) and by dynamic light scattering (Z-Average diameter) without indicating a polydispersity index (PDI). Purest Colloids provides size information for MesoSilver^®^ by dynamic light scattering (DLS).

Particle sizes (Z-Average, PDI) of the used nanomaterials were determined in water by DLS in a concentration of 100 ppm in disposable 70 μl micro UV cuvettes (BRAND GmbH, Wertheim, Germany). Analysis was performed on a Zetasizer Nano ZS (Malvern Instruments Ltd, UK) at 25°C (Software version 7.02). The dispersion medium was water exhibiting a viscosity of 0.8872 cP and a refractive index of 1.330. Stability of the samples in MEA liquid growth-medium was monitored by UV-Vis spectrophotometry over 7 days in disposable semi-micro UV cuvettes, layer thickness 10 mm (BRAND GmbH, Wertheim, Germany) exemplarily for 50 nm NanoComposix silica (1,000 ppm), 50 nm NanoComposix silver (10 ppm) and Purest Colloids MesoSilver^®^ (10 ppm). UV-Vis measurements were performed in triplicates using a Unicam UV1 spectrophotometer (Unicam Ltd, UK). Additionally photographs were taken for the same concentrations. For all methods the samples were not shaken and stored in the dark at 22°C +/- 2°C. Data are expressed as mean values ± standard deviation.

## Results and Discussion

### Characterization of nanomaterial

The sizes of the used nanoparticles in water were determined by dynamic light scattering (DLS) (Table 1). Purest Colloids specifies a size of typically 0.65 nm for the silver nanoparticles and states that MesoSilver^®^ is 0.9999 pure silver in colloidal form that contains only water and silver nanoparticles [20]. Furthermore a DLS measurement (size distribution report by volume) is provided (values are included in Table 1). The specified polydispersity index (0.827) at the report is very high. Values greater than 0.7, indicate that the sample has a very broad size distribution and is therefore not suitable for the DLS technique (Malvern 2011, Dynamic Light Scattering-Inform White Paper). As expected DLS in-house measurements did not meet its internal quality criteria and therefore we do not indicate a size for MesoSilver^®^ in Table 1.

**Fig 1:**
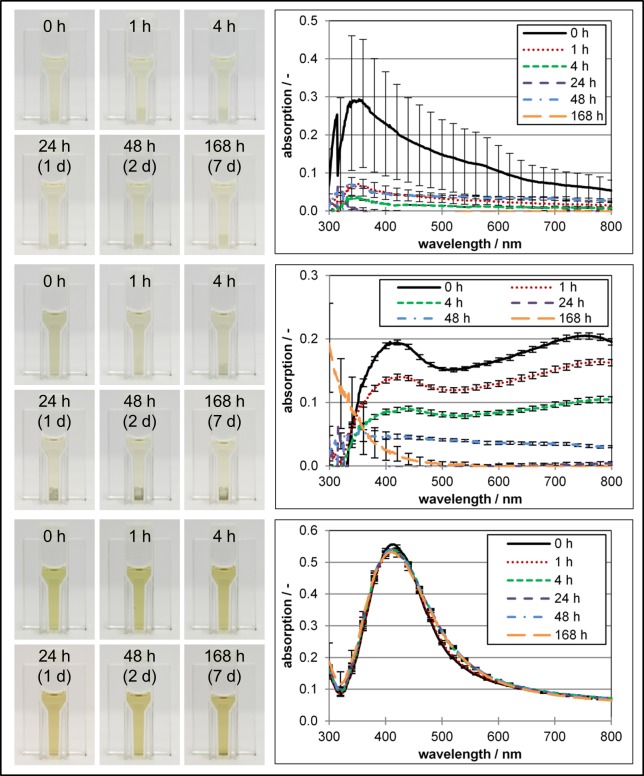
Stability of the nanoparticles in MEA (liquid) medium over 7 days. (a + b) NanoComposix silica (50 nm, 1,000 ppm). (c + d) NanoComposix silver (50 nm, 10 ppm). (e + f) Purest Colloids MesoSilver^®^ (10 ppm). Data in S1 data set.

After the size check in water the size and stability of the particles were investigated in MEA (liquid) medium (with Tween/NaCl/Agar) exemplarily for the 50 nm NanoComposix silica and silver particles as well as for the MesoSilver^®^. The 50 nm NanoComposix silica (1,000 ppm) and NanoComposix silver particles (10 ppm) were not stable in MEA medium (Fig 1A–1D). Therefore it was not possible to measure these samples by DLS. Formation of a deposition in the cuvettes was easily observable (Fig 1A–1C). This sedimentation of particles was verified by UV-Vis spectrophotometry. A decrease in absorption over time was observed (Fig 1B–1D). The high standard deviations especially for silica (t = 0 h) were due to the fast sedimentation of particles within the time of measurement. It has to be noted that the samples were not shaken during the stability analysis but they were in motion throughout the whole experiments ensuring that during the experimental procedure the particles were not able to settle down. Hence the particles have had contact to the mycelium over the whole incubation period. MesoSilver^®^ (10 ppm) was stable in MEA medium over 7 days (Fig 1E–1F). UV-Vis spectra did not change at all. Only a very slight black deposition on the bottom of the cuvette was observed.

### Effect of SiO_2_ nanomaterial on growth of *P*. *verrucosum*

Experiments started with supplementations of the growth medium using different sizes of SiO_2_ nanoparticles (20 nm, 50 nm, 100 nm, 200 nm). The selected concentration range was zero to 2,500 ppm (Table 1) reflecting a food relevant range. For instance, SiO_2_ (E551) is authorized within the European Union for use in foods in dried powdered form up to 10 g/kg equivalent to 10,000 ppm. Silver (E174) is authorized *quantum satis* for some products e.g. for liqueurs, as external coating of confectionery and for decoration of chocolates [21].

**Fig 2:**
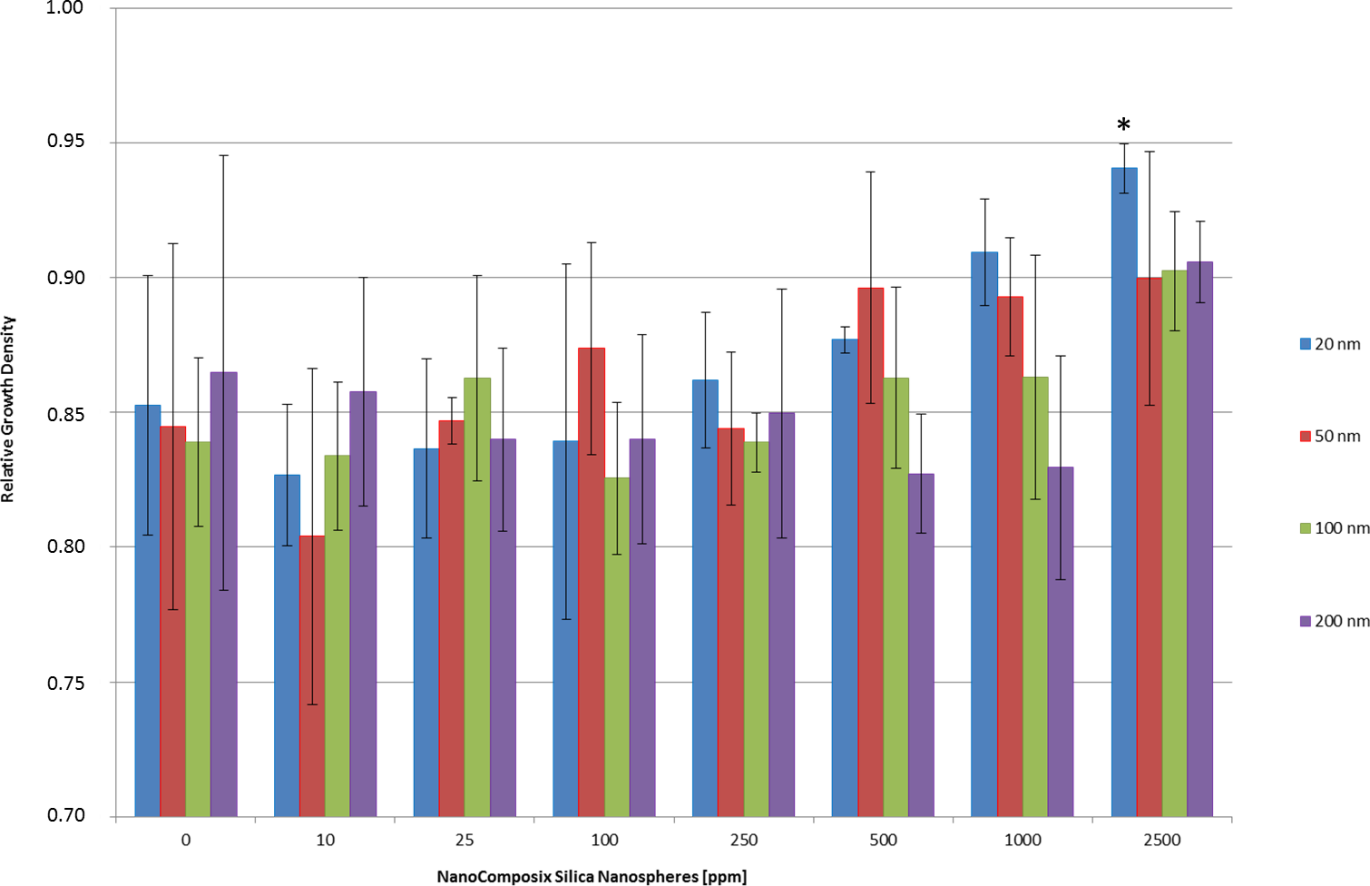
Relative growth density reflecting the growth rates of *Penicillium verrucosum* supplemented by NanoComposix SiO_2_ nanospheres. *Penicillium verrucosum* incubated 7 days at 25°C under shaking of 230 rpm in MEA-liquid medium, supplemented by different amounts (0–2,500 ppm) of NanoComposix SiO_2_ nanospheres exhibiting sizes of 20 nm, 50 nm, 100 nm and 200 nm. Significant differences (P < 0.05) are indicated with an asterisk. The growth rate is specified as relative growth density. Data in S2 data set.

The growth rates of *P*. *verrucosum* differ substantially in dependence of the size and the applied nanoparticle concentration (Fig 2). In detail, all particle-sizes showed to some extent a marginal growth-inhibiting effect at 10 ppm and in case of individual sizes up to a concentration of 250 ppm. Particles of 200 nm in size inhibited the fungal growth up to a concentration of 1,000 ppm. Interestingly, at 2,500 ppm the opposite was true and led to a strong increase in the growth rate. Particles of 20, 50 and 100 nm led to an increase in the growth rate at concentrations above 500 ppm. The smallest particles in size (20 nm) had the strongest growth supporting effect above 500 ppm. Particles of smaller sizes had in general and over the whole experimental setups a more pronounced influence on the growth rate in comparison to particles exhibiting larger sizes. This observed phenomenon, that different concentrations of nanoparticles could have indeed either a supporting or inhibiting effect on the physiological activity and growth of an organism, has been described earlier e.g. in case of plant cells [22–23]. The mechanism behind these observations is functionally analogue to diauxic growth in case of different available nutrients, where targeted biological answers, like e.g. the activation of alternative pathways, are induced after exceeding a defined physiological threshold value. Thus, different concentrations of supplemented nanoparticles result in the activation of diverse stress-compensation-pathways. Another factor is the varying and size-dependent interaction between the surface of the respective nanoparticle and the cell-wall which has e.g. an influence on the diffusion of nutrients and therefore affects also the growth rate.

### Effect of SiO_2_ nanoparticles on mycotoxin biosynthesis of *P*. *verrucosum*

**Fig 3:**
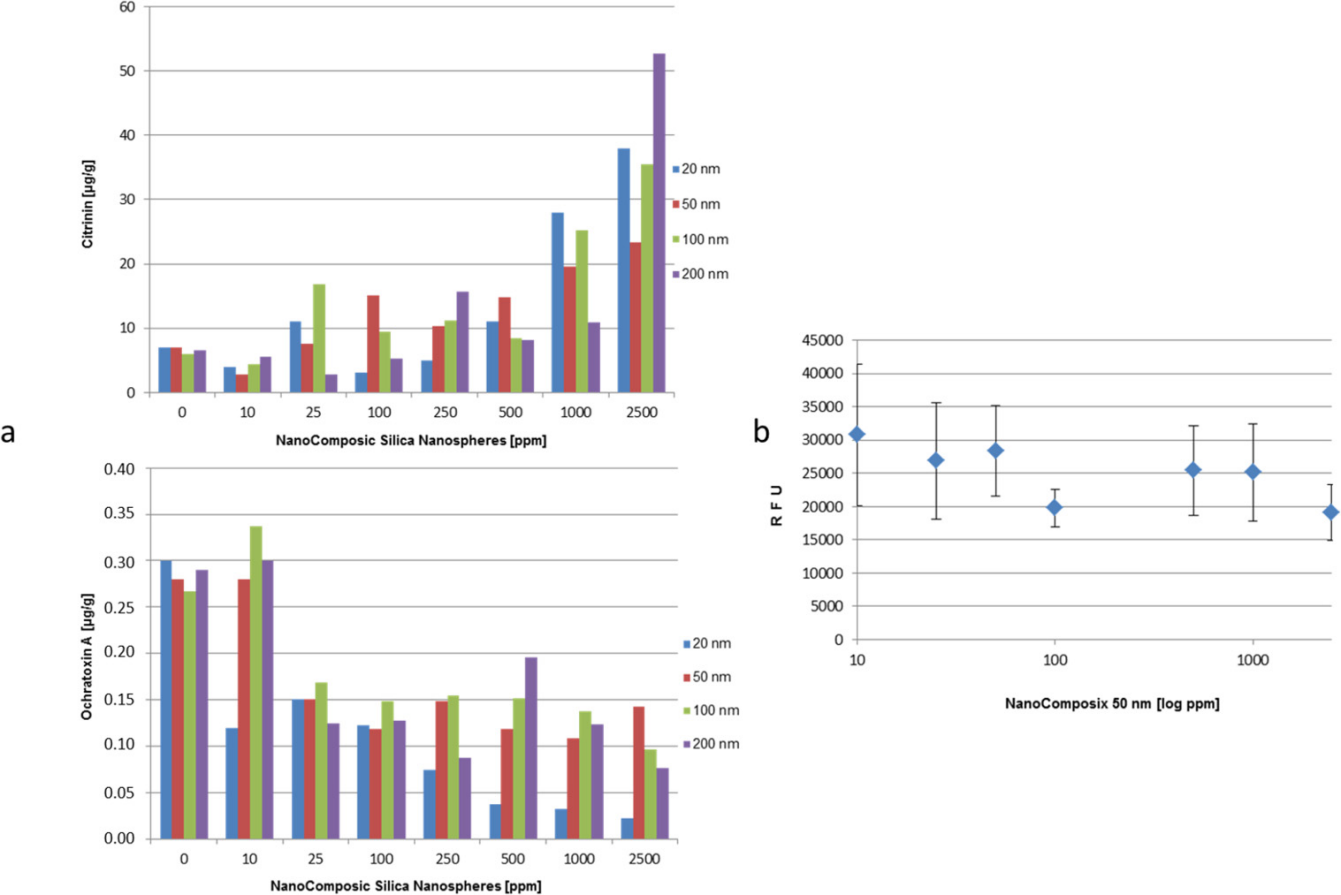
Mycotoxin biosynthesis and intracellular ROS level of *P*. *verrucosum* supplemented by NanoComposix SiO_2_ nanospheres. ROS and mycotoxins have been analyzed at the same time points. (**a**) Biosynthesis of citrinin and ochratoxin A by *P*. *verrucosum* growing 7 days at 25°C under shaking at 230 rpm in MEA-liquid medium supplemented by different amounts (0–2,500 ppm) of NanoComposix SiO_2_ nanospheres exhibiting sizes of 20 nm, 50 nm, 100 nm and 200 nm. (**b**) Determination of intracellular ROS in *P*. *verrucosum* at the end of 7 days of growth at 25°C, under shaking at 230 rpm, in MEA-liquid medium, supplemented by different amounts (0–2,500 ppm) of NanoComposix 50 nm sized SiO_2_ nanospheres. Logarithmic calculation. Data in S3 data set.

Fungal growth is mostly undesirable in food products and in case that the growing fungus produces deleterious substances such as mycotoxins, the supposed concomitant hazardous effects on consumer’s health are of major interest and have to be avoided. Hence, we analyzed whether supplementation with nanoparticles has substantial effects on the biosynthesis of the mycotoxins ochratoxin A as well as citrinin in *P*. *verrucosum*. Fig 3A clearly shows that *P*. *verrucosum* produced increasing amounts of citrinin in supplemented growth medium. This effect was paralleled by a strong decrease in ochratoxin A biosynthesis as a function of the concentration of SiO_2_ nanoparticles which itself depended strongly on the nanoparticle-size. This observation makes sense as it has been recently described by Schmidt-Heydt et al. [24], that mycotoxin biosynthesis, and in this case the citrinin biosynthesis, is induced when the fungal cell is subjected to oxidative stress [19] [25–26]. In fact, measuring the ROS within fungal mycelium grown at different concentrations of 50 nm nanoparticles draws the picture that the higher the concentration of 50 nm particles the lower the oxidative stress, reflected by a lower amount of ROS in the mycelium (Fig 3B). What appears contradictory at first glance, especially when keeping in mind that citrinin biosynthesis is assumed to be upregulated when oxidative stress increases, makes sense in fact when taking into account that a greater amount of synthesized citrinin within and surrounding the fungal mycelium results in a higher extent of radical scavening capacity by the action of the citrinin molecule [27]. Thus, this leads consequently to a lower amount of ROS in total. As another example it has been described that, if cells are subjected to low amounts of oxidative stress, the subsequent induction of stress-compensation-mechanisms (which is also true for citrinin biosynthesis) leads to stress adapted cells which are able to grow better at suboptimal growth conditions, respectively they tolerate more stressful conditions in comparison to cells which were not pre-stressed [28–30].

Maybe the evolutionary reason for ROS induced citrinin biosynthesis in the typical wheat-contaminating ascomycete *P*. *verrucosum* is the compensation of plant/host-specific defense mechanisms which particularly are based on ROS (oxidative burst, hypersensitivity response mechanisms) [31].

The decline in ochratoxin A biosynthesis, simultaneously to the gain in citrinin biosynthesis, is reasoned by the fact that both metabolites use the same acetyl-coA precursors and thus have consequently to be mutually regulated at the expense of each other. This has been recently described vice versa for growth on NaCl-rich substrates [32]. Hence, keeping in mind that animal and fungi share largely the same cell-architecture and predominantly signaling pathways are highly conserved within eukaryotic cells [33], it could be supposed that certain nanoparticles could be able to induce oxidative stress as well in human, animal and plant cells.

In summary, SiO_2_ nanoparticles were able to promote the growth of the analyzed filamentous fungi at higher concentrations. Furthermore, the citrinin biosynthesis of *P*. *verrucosum* was induced on cost of the ochratoxin A production. The extent of this induction depended on the concentration and the size of the respective nanoparticles.

### Effect of silver nanoparticles on the physiology of *P*. *verrucosum*

The main objective of this study was to investigate whether it is possible to substantially inhibit fungal growth by using nanoparticles which are already in daily-use. Therefore experiments were made with nanoparticles of another chemical class, in detail silver nanoparticles. It is a well-known fact that silver ions and silver particles, the latter especially in the nano-scale, exhibit bactericidal effects [34]. The hypothetical mechanism behind this observation is that nanoparticles whether as elemental silver or dissociated in Ag^+^-ions penetrate into microbial cells or are internalized by them and disturb essential cellular transport systems. These accumulated Ag^+^-ions/particles in turn interfere with the respiratory chain respectively important electron-transport-systems and lead as a consequence to the production of reactive oxygen species causing damage to proteins, lipids and nucleic acids [35–36].

**Fig 4:**
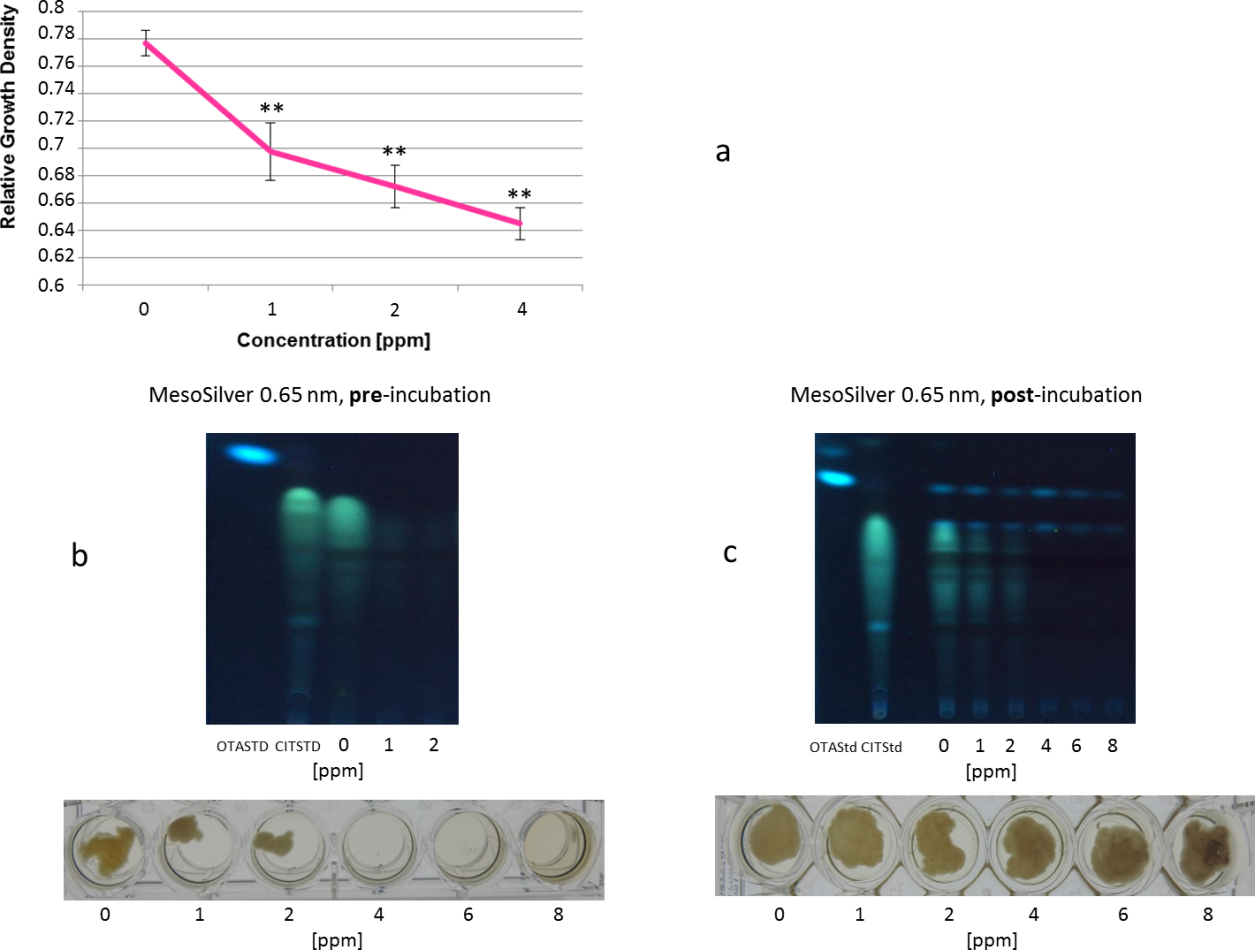
Relative growth density reflecting the growth rates and mycotoxin biosynthesis of *P*. *verrucosum* supplemented with sub-nano sized MesoSilver (0.65 nm). (**a**) Growth rate of *P*. *verrucosum* BFE575 at different concentrations of sub-nano sized MesoSilver (0.65 nm) 7 days at 25°C under rotation of 230 rpm in MEA-liquid medium. (**b**) Growth and mycotoxin biosynthesis visualized by TLC of *P*. *verrucosum* growing 7 days at 25°C under rotation of 230 rpm in MEA-liquid medium supplemented by different amounts of sub-nano sized MesoSilver (0.65 nm). MesoSilver has been applied **before** (!) growth of *P*. *verrucosum* (**c**). Growth and mycotoxin biosynthesis visualized by TLC of *P*. *verrucosum* growing 7 days at 25°C under rotation of 230 rpm in MEA-liquid medium supplemented by different amounts of sub-nano sized MesoSilver (0.65 nm). MesoSilver has been applied **after** (!) growth of *P*. *verrucosum*. Significant differences (P < 0.001) are indicated with an asterisk. The growth rate is specified as relative growth density. Data in S4 data set.

**Fig 5:**
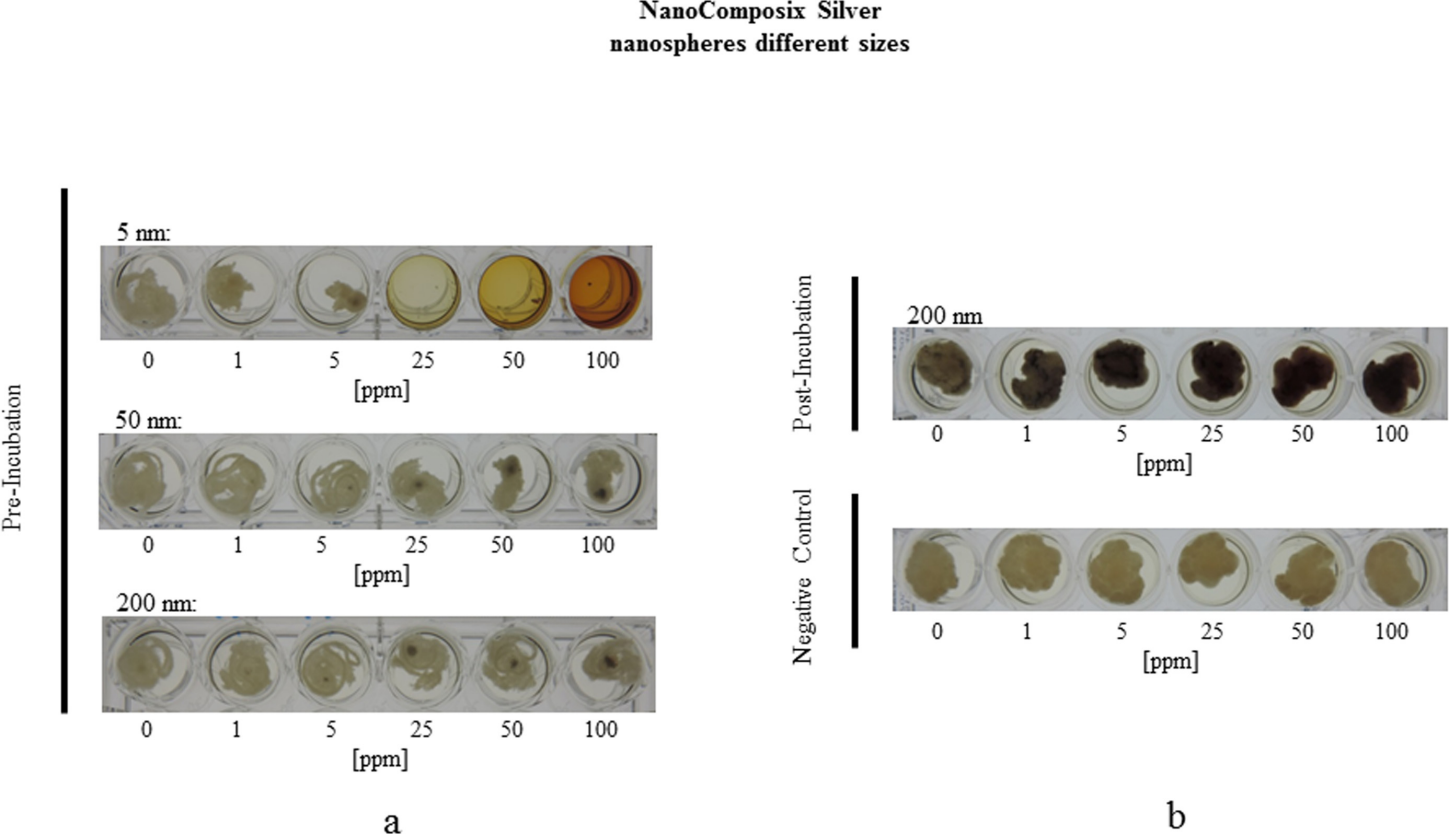
Growth morphology of *Penicillium verrucosum* supplemented by NanoComposix silver nanospheres. (a) Growth of *Penicillium verrucosum* 7 days at 25°C under rotation of 230 rpm in MEA-liquid medium supplemented by different amounts (0–100 ppm) of NanoComposix **silver** nanospheres exhibiting sizes of 5 nm, 50 nm and 200 nm. Silver has been applied **before** (!) growth of *P*. *verrucosum* (b). Growth of *Penicillium verrucosum* 7 days at 25°C under rotation of 230 rpm in MEA-liquid medium either as control or supplemented by different amounts (0–100 ppm) of NanoComposix **silver** nanospheres exhibiting a size of 200 nm. Silver has been applied **after** (!) growth of *P*. *verrucosum*.

**Fig 6:**
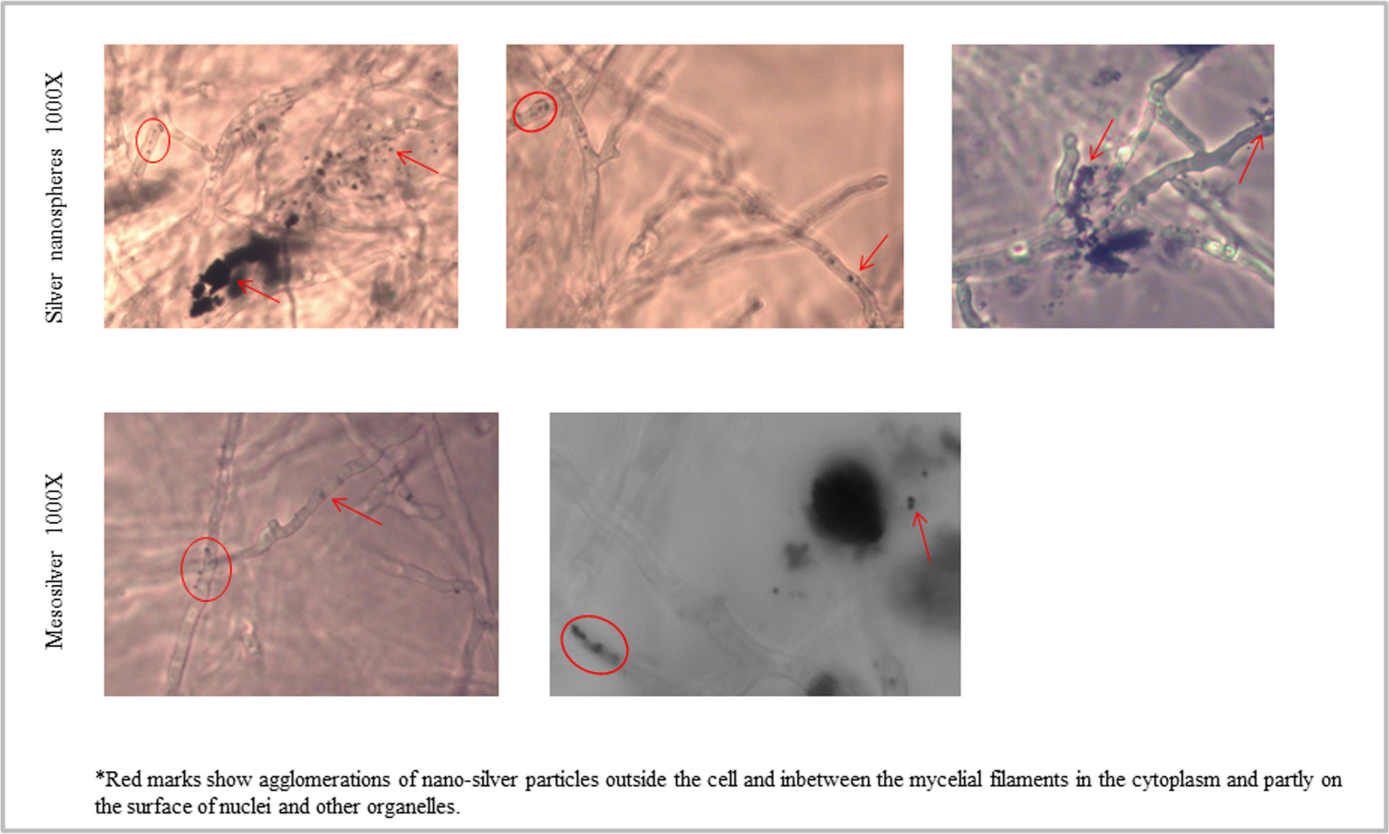
Microscopical examination of *P*. *verrucosum;* grown with 5 nm NanoComposix silver nanospheres or MesoSilver with 0.65 nm. Incubation was for 7 days at 25°C under rotation of 230 rpm. Magnification was 1000x using oil-immersion. Red marks show agglomerations of silver nanoparticles outside the cell and within the mycelial filaments in the cytoplasm and partly on the surface of nuclei and other organelles.

Analogous to the experiments with SiO_2_-particles we used colloidal subnano sized silver particles (0.65 nm, MesoSilver^®^) in a concentration range of 0 to 8 ppm and silver particles of the sizes 5 nm, 50 nm and 200 nm (NanoComposix) in the concentration range 0 to 100 ppm (Table 1). To analyze whether it matters if the silver nanoparticles were applied before germination of the spores or during mycelial growth, we either supplemented the growth medium before adding the fungal spores and after the fungus has grown 7 days. Fig 4A shows that colloidal silver nanoparticles, if applied before germination of the spores, led to a strong inhibition of germination and subsequent fungal growth. *P*. *verrucosum* was not able to grow at a silver nanoparticle concentration above 2 ppm. At 1 ppm to 2 ppm a weakened growth was observable, paralleled by a sharp decrease in citrinin biosynthesis (Fig 4B). Interestingly, if colloidal silver nanoparticles were applied after the fungus has already grown 7 days at 25°C, the fungus was only marginally affected and proceeded to grow over the whole concentration range (Fig 4C). In this case a substantial effect could be observed only for citrinin biosynthesis, which showed a clear decrease in a linear relationship to the concentration of supplemented silver nanoparticles. These findings imply that especially the germination of the spores was strongly inhibited but the growth of an already existing mycelium was nearly not affected. It could be clearly seen that small-sized silver particles (≤ 5nm) either penetrated the mycelial filaments or were ingested by them. Additionally, both, small-sized (0.65 nm, 5 nm) nanoparticles and nanoparticles exhibiting sizes above 5 nm (50 nm and 200 nm) attached strongly to the cell-wall of the mycelium (Fig 5) visualized as black shapes at concentrations above 2 ppm. Fig 5A shows that the 5 nm silver nanoparticles inhibited fungal growth and germination at concentrations above 5 ppm. For nanoparticles with a size of 50 nm the fungal growth was only weakly inhibited dependent of the concentration in a linear function. For 200 nm particles *P*. *verrucosum* was grown nearly unaffected up to 100 ppm. Again, (black-colored) silver nanoparticle agglomerates were attached to the mycelium. Furthermore, in every cavity were the fungus was able to grow, the specific coloration of the liquid-medium containing silver nanoparticles, reasoned by a physical effect called “surface plasmon resonance”, disappeared. Hence, the agglomeration and subsequent attachment of the particles to the fungal mycelium leads to an elimination of the silver nanoparticles from the liquid growth medium. This effect could be used for possible applications. In cases were the fungus could not grow based on total inhibition, the typical plasmon resonance caused coloration persists. This observation was supported by microscopical analyses of the mycelia at 400x to 1000x magnification. On Fig 6 it could be seen that the silver nanoparticles were attached to the mycelial cell surface and to some extend could also penetrate the fungal filaments resulting in silver agglomerates localized within the cytoplasm. Whether the nanoparticles are internalized by the cell or permeated because of their small size or/and as e.g. Ag^+^-ions, could not be assured and needs further investigations. Nevertheless, the effectivity in inhibiting the growth of *P*. *verrucosum* in case of small sized nanoparticles (5 nm; 0.65 nm) in comparison to the substantial less effective larger sized nanoparticles, gives a strong indication for our hypothesis that the small particles penetrate into the fungal cell and disturb important cellular mechanisms, whereas the larger did not. Similar observations have been reported recently by Bundschuh et al (2012), in this case with *Daphnia pulex*. Even low concentrations of nanoparticles (< 2 mg/l) showed significant interactions between the chitin exoskeleton of the water fleas [37]. In detail, the nanoparticles strongly bound to the chitin-tissue causing harmful effects. Furthermore, also the following generation of *Daphnia* showed anatomical impairments. These results strongly support our microscopical observations of a tight attachment of nanoparticle agglomerates to the mycelial surface, too. This makes sense, keeping in mind that the fungal cell wall consists of chitin as well. Thus, the use of nanoparticles should be reconsidered very carefully because it is likely that other eukaryotic cells are similar affected by some nanoparticles formulations, even if they are not consisted of chitin, because the mechanism behind this effect may be driven additionally by electrostatic interplay followed by cellular endocytosis [38–39].

Taken together, silver particles with a nominal size of 0.65 nm inhibit the germination of *P*. *verrucosum* at concentrations > 2 ppm. Nanoparticles with a size of 5 nm inhibit fungal growth above concentrations of 5 ppm and larger particles (50 nm, 200 nm) lead only to a weak inhibition over the whole concentration range. Furthermore, silver particles attach to the mycelial surface in cases where the fungus was able to germinate and grow (5a) and whether the nanoparticles are applied after the fungus has been already grown (Figs 4C and 5B). Microscopical examination of agglomerates localized within the cytoplasm as well as attached to cell nuclei strongly supports these observations (Fig 6); some agglomerates were localized outside the mycelium. Our findings confirm similar results of Hassan et al. [40]. The authors showed by Scanning Electron Microscopy (SEM) that silver nanoparticles attach to the cell membrane and may penetrate into fungal cells as well. Since nanoparticles have an extremely large surface area in comparison to their volume and a small size, the formation of Ag^+^-ions in case of silver is supported which could further precipitate in interaction with components of the growth media or fungal filaments. Thus consequently, their potential contact area with microorganisms like bacteria or fungi is substantially increased as well as the likelihood and the ability to permeate into cells. Therefor the effectivity of using nanoparticles in order to inhibit fungal growth and mycotoxin biosynthesis depends strongly on the chemical composition, the size and the concentration of the nanomaterial.

The large increase in the number of antibiotic-resistant bacteria and mycotoxigenic fungi has prompted a renewed interest in the development of alternatives to already established antibacterial and antifungal agents [41]. It could be proposed that the application of nanoparticles e.g. as spray-disinfectant could be a successful opportunity to prevent contaminations of food and feed caused by mycotoxigenic fungi. Thus, it may be feasible to use nanoparticle-based formulations as alternative treatment against fungal infections in human, animal, and plants. It has been described by Mittal et al. [42] that under certain conditions nanoparticles are able to penetrate the epidermis and serve as shuttle for human and animal vaccines. It should be considered, that the same mechanism could be used to transport fungicides into living plants. If the prolonged use of silver nanoparticles for inhibition of fungal growth could lead to the formation of resistant strains needs further investigations.

## Conclusion

Within this study it could be shown that nanoparticles could be used to inhibit the growth of mycotoxigenic fungal species like e.g. *P*. *verrucosum*. Nethertheless, with respect to the observed side-effects, the application of nanoparticles should be carefully reconsidered for each case, especially for food products.

## Supporting Information

S1 Data Dataset for Fig 1. Stability of the nanoparticles in MEA (liquid) medium over a time of 7 days.
(XLSX)

S2 Data Dataset for Fig 2. Growth rates of *Penicillium verrucosum* supplemented by NanoComposix SiO2 nanospheres.
(DOCX)

S3 Data Dataset for Fig 3. Mycotoxin biosynthesis and intracellular ROS level of *P*. *verrucosum* supplemented by NanoComposix SiO2 nanospheres.
(DOCX)

S4 Data Dataset for Fig 4. Growth rates and mycotoxin biosynthesis of *P*. *verrucosum* supplemented with sub-nano sized MesoSilver (0.65 nm).
(DOCX)

S5 Data Dataset for Table 1. Specifications of the applied nanomaterials.
(XLSX)
