# Supplementary material for: Influence of Different Nanomaterials on Growth and Mycotoxin Production of Penicillium verrucosum
Source: PLoS One. 2016 Mar 14;11(3):e0150855. doi: 10.1371/journal.pone.0150855 (PMC4790900; doi:10.1371/journal.pone.0150855)
Supplement: S2 Data — Growth rates of Penicillium verrucosum supplemented by NanoComposix SiO2 nanospheres. (DOCX) [file pone.0150855.s002.docx]

**S2 Growth rates of *Penicillium verrucosum* supplemented by NanoComposix SiO_2_ nanospheres.**

|  | PPM | Mean Values | | | Reciprocal Mean Values (= Relative Growth Density) | | | Average of Reciprocal Means (= Relative Growth Density) | Standard Deviation |
| --- | --- | --- | --- | --- | --- | --- | --- | --- | --- |
|  |  | #1 | #2 | #3 | #1 | #2 | #3 |  |  |
| NanoComposix 20 nm | 0 | 110 | 126.3 | 116.7 | 0.90909 | 0.79177 | 0.85690 | 0.85258 | 0.047995 |
|  | 10 | 125.8 | 121 | 116.4 | 0.79491 | 0.82645 | 0.85911 | 0.82682 | 0.026208 |
|  | 25 | 126.3 | 118.1 | 114.8 | 0.79177 | 0.84674 | 0.87108 | 0.83653 | 0.033175 |
|  | 100 | 133.3 | 110.2 | 116.3 | 0.75019 | 0.90744 | 0.85985 | 0.83916 | 0.065844 |
|  | 250 | 120.6 | 112.3 | 115.5 | 0.82919 | 0.89047 | 0.86580 | 0.86182 | 0.025177 |
|  | 500 | 114.7 | 114.2 | 113.2 | 0.87184 | 0.87566 | 0.88339 | 0.87696 | 0.004806 |
|  | 1000 | 109.1 | 113.3 | 107.6 | 0.91659 | 0.88261 | 0.92937 | 0.90952 | 0.019731 |
|  | 2500 | 105 | 107.5 | 106.5 | 0.95238 | 0.93023 | 0.93897 | 0.94053 | 0.009109 |
| NanoComposix 50 nm | 0 | 107.1 | 130 | 120.3 | 0.93371 | 0.76923 | 0.83126 | 0.84473 | 0.06782 |
|  | 10 | 129 | 134 | 112.3 | 0.77519 | 0.74627 | 0.89047 | 0.80398 | 0.06229 |
|  | 25 | 116.4 | 118.9 | 119 | 0.85911 | 0.84104 | 0.84034 | 0.84683 | 0.00869 |
|  | 100 | 113.8 | 108.8 | 121.5 | 0.87873 | 0.91912 | 0.82305 | 0.87363 | 0.03939 |
|  | 250 | 119.6 | 122.9 | 113.4 | 0.83612 | 0.81367 | 0.88183 | 0.84387 | 0.02836 |
|  | 500 | 105.1 | 118.1 | 112.3 | 0.95147 | 0.84674 | 0.89047 | 0.89623 | 0.04295 |
|  | 1000 | 116 | 109.8 | 110.4 | 0.86207 | 0.91075 | 0.90580 | 0.89287 | 0.02187 |
|  | 2500 | 110.9 | 104.6 | 118.9 | 0.90171 | 0.95602 | 0.84104 | 0.89959 | 0.04696 |
| NanoComposix 100 nm | 0 | N/A | 123.8 | 114.9 | N/A | 0.80775 | 0.87032 | 0.83904 | 0.03128 |
|  | 10 | 122.4 | 123.2 | 114.6 | 0.81699 | 0.81169 | 0.87260 | 0.83376 | 0.02755 |
|  | 25 | 115.7 | 122.7 | 110.1 | 0.86430 | 0.81500 | 0.90827 | 0.86252 | 0.03810 |
|  | 100 | 126.2 | 116.1 | 121.5 | 0.79239 | 0.86133 | 0.82305 | 0.82559 | 0.02820 |
|  | 250 | 121 | 117.2 | 119.5 | 0.82645 | 0.85324 | 0.83682 | 0.83884 | 0.01103 |
|  | 500 | 109.9 | 119.9 | 118.4 | 0.90992 | 0.83403 | 0.84459 | 0.86285 | 0.03356 |
|  | 1000 | 110 | 113.8 | 124.8 | 0.90909 | 0.87873 | 0.80128 | 0.86304 | 0.04539 |
|  | 2500 | 113.8 | 107.3 | 111.5 | 0.87873 | 0.93197 | 0.89686 | 0.90252 | 0.02210 |
| NanoComposix 200 nm | 0 | 102 | 125 | 123 | 0.97847 | 0.80257 | 0.81301 | 0.86468 | 0.08057 |
|  | 10 | 117 | 124 | 110 | 0.85470 | 0.80710 | 0.91075 | 0.85752 | 0.04236 |
|  | 25 | 117 | 126 | 115 | 0.85690 | 0.79239 | 0.87032 | 0.83987 | 0.03402 |
|  | 100 | 126 | 120 | 112 | 0.79491 | 0.83542 | 0.88968 | 0.84000 | 0.03882 |
|  | 250 | 120 | 110 | 124 | 0.83056 | 0.91324 | 0.80515 | 0.84965 | 0.04615 |
|  | 500 | 117 | 124 | 122 | 0.85763 | 0.80580 | 0.81833 | 0.82726 | 0.02208 |
|  | 1000 | 118 | 116 | 130 | 0.85106 | 0.86580 | 0.77160 | 0.82949 | 0.04137 |
|  | 2500 | 110 | 109 | 113 | 0.91158 | 0.92081 | 0.88496 | 0.90578 | 0.01520 |
