# Supplementary material for: Influence of Different Nanomaterials on Growth and Mycotoxin Production of Penicillium verrucosum
Source: PLoS One. 2016 Mar 14;11(3):e0150855. doi: 10.1371/journal.pone.0150855 (PMC4790900; doi:10.1371/journal.pone.0150855)
Supplement: S3 Data — Mycotoxin biosynthesis and intracellular ROS level of P. verrucosum supplemented by NanoComposix SiO2 nanospheres. (DOCX) [file pone.0150855.s003.docx]

**S3A Table. Mycotoxin biosynthesis of *P. verrucosum* supplemented by NanoComposix SiO_2_ nanospheres.**

|  | PPM | Citrinin [µg/µl] | Ochratoxin A [µg/µl] |  | PPM | Citrinin [µg/µl] | Ochratoxin A [µg/µl] |
| --- | --- | --- | --- | --- | --- | --- | --- |
| NanoComposix 20 nm | 0 | 7 | 0.3 | NanoComposix 100 nm | 0 | 6 | 0.267 |
|  | 10 | 4 | 0.12 |  | 10 | 4.438 | 0.337 |
|  | 25 | 11 | 0.151 |  | 25 | 16.793 | 0.169 |
|  | 100 | 3 | 0.123 |  | 100 | 9.403 | 0.149 |
|  | 250 | 5 | 0.074 |  | 250 | 11.217 | 0.155 |
|  | 500 | 11 | 0.037 |  | 500 | 8.469 | 0.152 |
|  | 1000 | 28.00 | 0.03 |  | 1000 | 25.147 | 0.138 |
|  | 2500 | 38 | 0.022 |  | 2500 | 35.423 | 0.096 |
| NanoComposix 50 nm | 0 | 7 | 0.28 | NanoComposix 200 nm | 0 | 6.5 | 0.29 |
|  | 10 | 2.797 | 0.28 |  | 10 | 5.547 | 0.3 |
|  | 25 | 7.613 | 0.151 |  | 25 | 2.753 | 0.125 |
|  | 100 | 15.077 | 0.119 |  | 100 | 5.225 | 0.128 |
|  | 250 | 10.236 | 0.149 |  | 250 | 15.727 | 0.087 |
|  | 500 | 14.723 | 0.119 |  | 500 | 8.185 | 0.196 |
|  | 1000 | 19.557 | 0.108 |  | 1000 | 10.879 | 0.124 |
|  | 2500 | 23.323 | 0.143 |  | 2500 | 52.743 | 0.076 |

**S3B Table. Intracellular ROS level of *P. verrucosum* supplemented by NanoComposix SiO_2_ nanospheres.**

| NanoComposix 50 nm [ppm] | # 1 | # 2 | Mean | Standard Deviation |
| --- | --- | --- | --- | --- |
| 0 | 26051 | 39561 | 32806 | 9553 |
| 10 | 23285 | 38340 | 30813 | 10645 |
| 25 | 20653 | 33108 | 26881 | 8807 |
| 100 | 23537 | 33151 | 28344 | 6798 |
| 250 | 17821 | 21838 | 19830 | 2840 |
| 500 | 20669 | 30181 | 25425 | 6726 |
| 1000 | 19980 | 30308 | 25144 | 7303 |
| 2500 | 16129 | 22074 | 19102 | 4204 |
