# Supplementary material for: Influence of Different Nanomaterials on Growth and Mycotoxin Production of Penicillium verrucosum
Source: PLoS One. 2016 Mar 14;11(3):e0150855. doi: 10.1371/journal.pone.0150855 (PMC4790900; doi:10.1371/journal.pone.0150855)
Supplement: S4 Data — Growth rates and mycotoxin biosynthesis of P. verrucosum supplemented with sub-nano sized MesoSilver (0.65 nm). (DOCX) [file pone.0150855.s004.docx]

**S4 Table. Growth rates *P. verrucosum* supplemented with sub-nano-sized MesoSilver (0.65 nm).**

|  | PPM | Mean Values | | | | | Reciprocal Mean Values (= Relative Growth Density) | | | | | Average of Reciprocal Means (= Relative Growth Density) | Standard Deviation |
| --- | --- | --- | --- | --- | --- | --- | --- | --- | --- | --- | --- | --- | --- |
|  |  | #1 | #2 | #3 | #4 | #5 | #1 | #2 | #3 | #4 | #5 |  |  |
| MesoSilver 0.65 nm | 0 | 127.9 | 127.3 | 128.2 | 128.3 | 131.7 | 0.78186 | 0.78555 | 0.78003 | 0.77942 | 0.75930 | 0.77712 | 0.00922 |
|  | 1 | 149 | 146.8 | 136.6 | 142.7 | 141.6 | 0.67114 | 0.68120 | 0.73206 | 0.70077 | 0.70621 | 0.69764 | 0.02116 |
|  | 2 | 150.5 | 151.8 | 142.5 | 151.1 | 148 | 0.66445 | 0.65876 | 0.70175 | 0.66181 | 0.67568 | 0.67213 | 0.01571 |
|  | 4 | 159.3 | 153.9 | 157.5 | 157.2 | 118.5 | 0.62775 | 0.64977 | 0.63492 | 0.63613 | 0.66000 | 0.64500 | 0.01159 |
